# Presentations of Children with Suspected Sepsis Caused by Acute Infectious Diarrhea in the Pediatric Emergency Department

**DOI:** 10.3390/children11020171

**Published:** 2024-01-29

**Authors:** Tai-An Lee, Chun-Yu Chen, Yu-Jun Chang, Bei-Cyuan Guo, Wen-Ya Lin, Chao-Hsin Wu, Han-Ping Wu

**Affiliations:** 1 Department of Emergency Medicine, Chang Bing Show Chwan Memorial Hospital, Changhua 50544, Taiwan; diane.lee.em@gmail.com; 2 Department of Emergency Medicine, Tungs’ Taichung MetroHarbor Hospital, Taichung 43503, Taiwan; t14367@ms3.sltung.com.tw; 3 Department of Nursing, Jen-Teh Junior College of Medicine, Nursing and Management, Miaoli 35664, Taiwan; 4 Laboratory of Epidemiology and Biostastics, Changhua Christian Hospital, Changhua 50006, Taiwan; 83686@cch.org.tw; 5 Department of Pediatrics, National Cheng Kung University Hospital, College of Medicine, National Cheng Kung University, Tainan 70403, Taiwan; n109676@mail.hosp.ncku.edu.tw; 6 Department of Pediatric Emergency Medicine, Taichung Veteran General Hospital, Taichung 43503, Taiwan; wenya@vghtc.gov.tw; 7 Department of Pediatrics, Taichung Veteran General Hospital, Taichung 43503, Taiwan; 8 Department of Post-Baccalaureate Medicine, National Chung Hsing University, Taichung 40202, Taiwan; 9 College of Medicine, Chang Gung University, Taoyuan 33302, Taiwan; 10 Department of Pediatrics, Chiayi Chang Gung Memorial Hospital, Chiayi 61363, Taiwan

**Keywords:** acute infectious diarrhea, sepsis, emergency department, children

## Abstract

Background: Acute infectious diarrhea is a common cause of hospitalization in children. Hence, early identification of acute bacterial gastroenteritis with suspected sepsis in pediatric emergency departments (EDs) is important. This study aimed to describe the clinical spectrum and initial characteristics of children who were presented to a pediatric ED with acute infectious diarrhea and suspected sepsis. Methods: Between April 2020 to March 2021, children with clinical diagnoses of acute bacterial colitis and suspected sepsis who were admitted to the pediatric ED were prospectively enrolled. The following data were obtained and compared between different age groups of children: including demographics, presentation, laboratory tests, culture results, treatment modalities, complications, and short-term outcomes. Results: A total of 105 patients (70 males and 35 females; mean age: 3.75 ± 3.52 years) were enrolled in this study. Of them, 89 (84.8%) patients were <6 years of age, and 80 (76.2%) patients required hospitalization for a duration of 4.7 ± 2.08 days. C-reactive protein (CRP) and procalcitonin (PCT) levels were significantly higher in the admission (both *p* < 0.001) and anti-biotic treatment groups (both *p* < 0.001). *Salmonella enteritidis* was the most common organism cultured from the stool and blood samples (39 of 91 (38.5%) and 2 of 105 (1.9%), respectively). Conclusions: The primary causative organism of acute infectious diarrhea identified in this study was *S. enteritidis*. Age and elevated serum CRP or PCT levels could be important factors in the decisions of emergency physicians regarding hospitalization and antibiotic therapies for pediatric acute infectious diarrhea.

## 1. Introduction

Acute infectious diarrhea is one of the most common illnesses in children. Most episodes are self-limiting and require only supportive treatment. However, acute infectious diarrhea may result in emergency visits and hospitalization, and remains the second leading cause of mortality in children <5 years of age worldwide [1,2,3]. Causative pathogens of pediatric acute infectious diarrhea are diverse and include viruses, bacteria, and protozoa. Viruses are the leading cause of pediatric acute infectious diarrhea; the two most common viruses are rotavirus and norovirus [4]. 

In Taiwan, the epidemiology of acute infectious diarrhea has changed over the past 15 years since the introduction of two rotavirus vaccines [5]. In a previous study, non-typhoidal *Salmonella* (NTS) was the most isolated bacterial enteric pathogen. *Campylobacter*, *Escherichia coli* 0157, *Shigella*, and *Yersinia enterocolitica* were the other pathogens isolated in children <5 years of age [6]. *Salmonella* species are the most common causative pathogens of pediatric bacterial enterocolitis in Taiwan, and are an important cause of bacterial enterocolitis worldwide [7].

Generally, the clinical presentation of bacterial gastroenteritis includes a sudden onset of fever, vomiting, abdominal cramps, diarrhea, headache, and myalgia [8]. However, the clinical severity of bacterial gastroenteritis may range from mild symptoms to serious complications, such as bacteremia, sepsis, and septic shock, particularly in very young and immunocompromised patients [9]. Sepsis is a potentially life-threatening clinical condition; hence, early recognition and prompt treatment are critical. A preceding study identified several predictors, including a younger age, dry and cold season, nutritional status characterized by increased height, absence of bloody diarrhea, and vomiting, as the clinical factors most indicative of viral etiology in cases of diarrhea [10]. Moreover, in the context of developing countries, when pediatric patients exhibit symptoms such as abdominal pain, fever, and bloody-mucous diarrhea, it is prudent to consider NTS as a potential causative agent [11]. However, it is difficult for emergency physicians to diagnose acute bacterial gastroenteritis early and provide accurate prognoses in children based solely on their clinical presentation during emergency department (ED) visits. 

The Vesikari Scoring System (VSS) is a severity scale that has been widely adopted and may be considered a useful tool for distinguishing between bacterial and viral pathogens in pediatric gastroenteritis [12,13]. This study aimed to analyze the clinical spectrum of children with acute febrile infectious diarrhea, suspected sepsis, and VSS scores ≥ 10 who presented at our pediatric emergency department (ED). Additionally, the study aimed to identify the initial clinical characteristics that could help emergency physicians to predict acute infectious diarrhea outcomes.

## 2. Materials and Methods

### 2.1. Patient Population

This was a single-center, prospective, observational study. Patients < 18 years of age diagnosed with acute febrile infectious diarrhea with suspected sepsis and VSS scores ≥ 10 admitted to the ED in a medical center in central Taiwan were enrolled from April 2020 to March 2021. During the 1-year study period, about 18,000 children presented to our pediatric ED. This study was approved by the Institutional Review Board and Ethics Committee of China Medical University Hospital (No. CMUH109-REC1-015). All methods were performed in accordance with relevant guidelines and regulations. Data were collected, reviewed, de-identified, and anonymized prior to analysis. Informed consent was obtained from all the guardians of the children who participated in this study.

### 2.2. Study Design

Children were enrolled according to the following inclusion criteria: (i) age between 1 month and 18 years, (ii) acute diarrheal symptoms within 1 week before the ED visit, (iii) fever (≥38 °C), (iv) suspected sepsis, (v) VSS scores ≥ 10, and (vi) clinical diagnosis of acute bacterial colitis by pediatric emergency physicians. The exclusion criteria were (i) inflammatory bowel disease (Crohn’s disease and ulcerative colitis), (ii) antimicrobial therapy administered 1 week before the ED visit, (iii) recent international travel (cross-border travel to a foreign country within 2 weeks before visit), and (iv) discharge diagnosis of urinary tract infection. 

Acute diarrhea was defined as symptoms of diarrhea (loose or watery stools > 3 times per day) within 1 week before ED presentation. Fever was defined as an ear temperature of ≥38 °C. Dehydration in this study was defined in accordance with the clinical dehydration scale, which considers four key clinical characteristics: general appearance, appearance of the eyes, hydration status of the mucous membranes (such as the tongue), and the presence of tears [14]. Each characteristic is assigned a score of 0, 1, or 2, resulting in a total score ranging from 0 to 8. A score of 0 indicates no dehydration, scores from 1 to 4 indicate some dehydration, and scores from 5 to 8 indicate moderate to severe dehydration [14]. Sepsis was defined as the presence of both a suspected or confirmed infection and systemic inflammatory response syndrome [15]. The treatment strategies were based on the severity of sepsis: (i) septic shock or sepsis associated organ dysfunction: administer a rapid 0.9% normal saline bolus at a dosage of 20 mL/kg and initiate antibiotic administration within 60 min, and (ii) no clinical shock or concern for sepsis-associated organ dysfunction: 0.9% normal saline bolus at a dosage of 20 mL/kg or administer antibiotics within 180 min if indicated.

**Figure 1:**
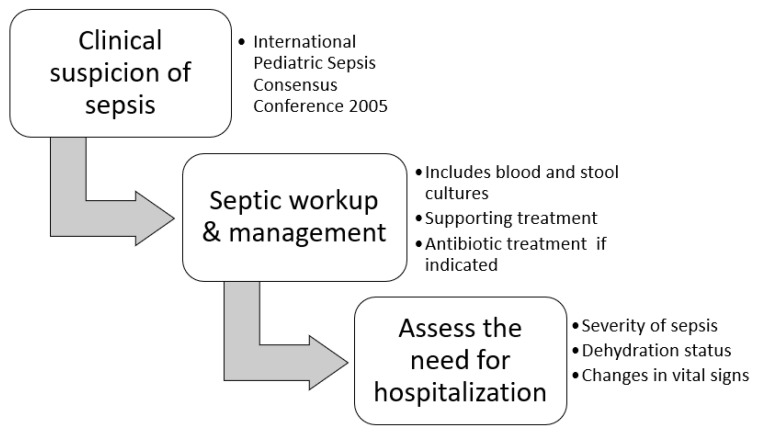
A straightforward flowchart outlining our research process in the ED.

The VSS assigns scores based on various symptoms and signs, and treatment modalities, including the duration and frequency of diarrhea, vomiting episodes, fever, dehydration, and hospitalization [12,13]. Higher scores on the VSS may indicate increased frequency and duration of vomiting or diarrhea, elevated body temperature, a more severe degree of dehydration, and an elevated risk necessitating admission for treatment. The clinical criteria for diagnosing acute bacterial colitis encompass a thorough evaluation of the patient’s medical history, physical examination, and laboratory investigations. The following information was obtained from the medical records of each patient: (i) basic demographic characteristics (i.e., age, sex, and pre-existing medical conditions), (ii) associated symptoms and signs (fever, diarrhea, vomiting, gross mucus or blood in stool, abdominal pain, dehydration, duration of fever, and diarrhea before admission), and (iii) laboratory tests (initial serum white blood cell (WBC), differential count, hemoglobin, electrolytes, C-reactive protein (CRP) level, procalcitonin (PCT) level, blood culture, and stool culture). Other clinical data, including the length of hospital stay, treatment modalities, complications, and long-term outcomes, were collected and analyzed. Furthermore, patients were divided into three age groups: (i) infants (≤1 year), (ii) young children (2–6 years), and older children (≥7 years). The variables were compared and analyzed among the different age and management groups. A straightforward flowchart depicting our research process in the ED is presented in Figure 1.

### 2.3. Statistical Analysis

In this study, continuous variables were presented as means ± standard deviation, whereas the categorical variables were presented as numbers and percentages. We first used univariate analysis to explore potential risk factors for hospitalization. Therefore, demographic and clinical data between hospitalized and non-hospitalized patients were compared using the chi-square test, Fisher’s exact test, or Mann–Whitney U test to identify factors with statistically significant differences. Logistic regressions were then used to predict factors associated with hospitalization. We selected independent variables with *p* values less than 0.05 in the univariate analysis to perform multivariate analysis and calculate the odds ratio of hospitalization. Only significant predictors (*p* < 0.05) were retained in the final model. All the data in this study were analyzed using the IBM SPSS Statistics for Windows, Version 22.0 (IBM Corp., Armonk, NY, USA). *p*-values < 0.05 were considered statistically significant.

## 3. Results

### 3.1. Demographics and Clinical Presentations of Patients with Acute Infectious Diarrhea

During the 1-year study period, 105 patients (70 males and 35 females; mean age, 3.75 ± 3.52 years; range, 4 months to 15 years old) who presented at the ED with clinical acute bacterial colitis were enrolled. Among all patients, 21 (20%) belonged to the infant age group, 68 (64.8%) were in the toddler and pre-school age group, and 16 (15.2%) were in the school and adolescent age group. The three most common symptoms and signs, except fever and diarrhea at presentation, were gross mucus in stool, vomiting, and abdominal pain (57.1%, 46.7%, and 41.9%, respectively; Table 1). Gross mucus and blood in the stool were the most common signs in infants. In contrast, the most common symptoms and signs detected in the older child group were chillness, abdominal pain, headache, and muscle soreness. 

Only two (1.9%) of the 105 patients had an underlying disease (biliary atresia and extremely preterm with development delay). Eighty (76.2%) of the 105 patients were treated upon admission, and complications of meningitis and osteomyelitis were not observed.

Laboratory evaluations showed a significant difference in platelet count, hemoglobin, potassium, and glucose levels between the different age groups (Table 2). Additionally, neutrophil and lymphocyte percentages differed significantly among the three groups. However, there are also differences in the blood formula of healthy children by age. Of the 105 patients with probable acute bacterial colitis, 93 (88.6%) of them presented with elevated CRP levels (normal range: <8 mg/L), and 38 (36.9%) of 103 children who obtained a complete blood count (CBC) presented with leukocytosis (WBC count: >10,000/μL). The mean WBC counts in these 103 patients were 9740 ± 4520/μL (range: 3700–25,000/μL). Moreover, the WBC count, PCT, and CRP levels did not significantly differ between the different age groups.

The duration of fever (2.56 ± 1.34 days) and diarrhea (2.54 ± 1.58 days) between onset and the pediatric ED visit was significantly longer in the young children group than in the other two age groups (*p* = 0.013 and *p* = 0.027, respectively). The mean duration of hospitalization in 80 patients was 4.7 ± 2.08 days, and the mean time to fever resolution after admission was 2.44 ± 1.75 days. The average duration of stay in the ED observation unit for 25 patients was 5.16 ± 2.73 h. No children were admitted to the intensive care unit, and there were no instances of mortality in our study.

### 3.2. Comparison of Demographics, Laboratory Tests, and Antibiotic Therapy between Hospitalized and Nonhospitalized Patients

The mean age of admitted patients was significantly younger than that of children discharged from the ED (3.08 ± 2.82 and 5.92 ± 4.61 years, respectively; *p* = 0.007). The differences in laboratory data between the patients with and without inpatient admission are listed in Table 3. CRP and PCT levels were significantly higher in the admission group than in the non-admission group (*p* < 0.001 and *p* < 0.001, respectively), and their mean levels were 67.6 ± 57.8 mg/L and 3.4 ± 8.5 ng/mL, respectively. In addition, the neutrophil and lymphocyte percentages significantly differed between these two groups (*p* = 0.037 and *p* = 0.028, respectively). However, it is well known that the blood formula of healthy children also varies with age. The results showed that age, CRP, and PCT levels were significant factors for the need for hospital admission. Multiple logistic regression analysis was then performed using the variables that had shown significant differences (*p* < 0.05) be-tween the admission and the non-admission groups in the univariate analysis. The findings revealed that three parameters were associated with an increased rate of admission: younger age, increased CRP and PCT levels (odds ratio, 1.404; *p* = 0.025 vs. odds ratio, 13.455; *p* = 0.043, respectively; Table 4).

Differences in laboratory data of admitted patients with and without antibiotic treatment are listed in Table 5. CRP and PCT levels were significantly higher in the antibiotic treatment group than in the group without antibiotic treatment (*p* < 0.001 and *p* < 0.001, respectively), and their mean levels were 85.8 ± 63.2 mg/L and 5.1 ± 10.3 ng/mL, respectively. Furthermore, the percentage lymphocyte and platelet counts were significantly different between these two groups (*p* = 0.006 and *p* = 0.01, respectively). Patients who required anti-biotic treatment had longer hospitalization durations than those without antibiotics treatment (*p* < 0.001). In total, 51 (63.8%) of the 80 hospitalized patients who required antibiotic treatment, included 42 cases of ceftriaxone, 8 cases of cefotaxime, and 1 case of piperacillin-tazobactam. All 17 isolates form these 51 patients were not resistant to third- and fourth-generation cephalosporins (ceftriaxone, cefotaxime, ceftizoxime, cefepime).

### 3.3. Comparison of Characteristics and Associations of Admitted Children Based on the Presence of S. enteritidis in Stool Culture

Of the 80 admitted patients, 30 (37.5%) had organisms identified in their stool or blood culture samples, and *S. enteritidis* was isolated from the stool samples of 28 children (Table 6). Four of the 80 (5%) patients had positive blood cultures, including two with *S. enteritidis*, one with *Staphylococcus aureus*, and one with *Janibacter hoylei.* Gross blood and mucus in stool were more common in the *S. enteritidis*-positive culture group than in the *S. enteritidis*-negative culture group; this difference was statistically significant (*p* = 0.018 and *p* = 0.016, respectively). Patients with positive stool culture results had a higher risk of pro-longed hospitalization (>5 days) than those with negative stool culture results (*p* = 0.024).

## 4. Discussion

Most acute infectious diarrhea episodes are self-limiting; however, colitis caused by various pathogenic bacteria may result in a potentially life-threatening clinical condition. During this study, most cases (84.8%) occurred in patients < 6 years of age, and the ratio of males to females was 2:1; this is similar to previous studies [8,9]. Seventy-five percent of childhood bacteremia occurred within the first 12 months of life, which is consistent with previous studies showing that NTS bacteremia in children occurs at a young age, especially in infancy [16,17]. In this, all children presented with fever and diarrhea, and 49%, 44%, and 37% presented with vomiting, abdominal pain, and cold, respectively. Moreover, cold, abdominal pain, headache, and muscle soreness were the most common symptoms and signs detected in the older children group. In addition, gross mucus was more common than gross blood in stool, and both occurred at significantly higher rates in the infant group than in the other groups. These findings suggest that the clinical presentation of acute febrile infectious colitis varies among different age groups. Also, it is difficult to predict stool culture results based on clinical symptoms and signs alone.

No specific laboratory tests for diagnosing acute bacterial colitis exist. In the present study, the most common abnormal laboratory data included CRP levels > 8 mg/L (88.6%) and PCT levels > 0.5 ng/mL (48.5%). Among the children, 36.9%, 25.7%, 8.7%, and 2.9% presented with leukocytosis (WBC count: >10,000/μL), hyponatremia (serum sodium: ≤135 mEq/L), hypokalemia (serum potassium: ≤3.5 mEq/L), and hypoglycemia (serum glucose: ≤70 mg/dL), respectively. Only one child had severe hypoglycemia (serum glucose: ≤54 mg/dL), and severe hyponatremia or hypokalemia was not observed in any children. 

A previous study reported by Park et al. has indicated that CRP at a cut-off value of 13.7 mg/L showed moderate diagnostic sensitivity (83.3%) and specificity (68.2%) with a 29% prevalence of bacterial diarrhea [18]. In our current study, 85 (81%) of 105 patients had CRP levels higher than13.7 mg/L. Of the 80 admitted patients, 30 (37.5%) had organisms identified in their stool or blood culture samples, and 27 (90%) of 30 patients had CRP levels above 13.7 mg/L, and 15 (50%) of 30 patients had PCT levels above or equal 0.5 ng/mL. Based on these results, it is recommended that emergency physicians should consider a diagnosis of acute bacterial colitis when children present with fever, diarrhea, and elevated serum CRP or PCT levels, even without presenting with leukocytosis.

The overall admission rate in this study was 76.2%, and the mean age of the admitted children was significantly younger than that of those discharged from the ED. The mean duration of hospitalization in these children was 4.7 ± 2.08 days and was shorter than durations reported in other studies in Taiwan that included hospitalized children diagnosed with S. enterocolitis [19,20].

Many studies have focused on the characteristics of viral gastroenteritis and the etiology, epidemiology, and clinical features of pediatric patients hospitalized due to AGE [5,6,8,9,19,20,21,22]. However, relatively few comprehensive studies have focused on admission indicators in children presenting with acute infectious diarrhea in the ED. This study found that age and serum CRP and PCT levels may be important factors to indicate that admission may be required for children presenting to the ED with fever, VSS ≥ 10, and acute infectious diarrhea. 

In total, 39 of 91 (42.9%) patients had positive stool culture results while 32 of 76 (42.1%) admitted patients had positive stool cultures in this study. The overall positive blood culture rate was 3.8%, and *S. enteritidis* was the most common organism cultured from the stool and blood samples. In addition, two patients < 2 years of age had NTS bacteremia (serogroups B and C2), and one had negative stool culture results. This study’s findings were similar to those of a previous study published by Hung et al. in 2017. They reported that NTS bacteremia occurred in 3% of patients, and Salmonella serogroup C2 was the most common group [8]. In this study, the positive rate of stool cultures decreased with age, and macroscopic blood and mucus in the stool might have significantly increased the probability of *S. enteritidis*-positive cultures. In addition, children admitted with positive stool cultures had an increased duration of hospitalization > 5 days. 

Based on previous studies, recommended therapies for acute infectious gastroenteritis, NTS gastroenteritis, and invasive NTS vary significantly in children [9,23,24,25,26,27]. Intravenous administration of cephalosporins (ceftriaxone or cefotaxime) is recommended as the first-line empirical treatment for children with serious NTS infections [8,22,25,27]. However, the distribution of serogroups and antimicrobial resistance among NTS isolates from children has changed over the past two decades in Taiwan [8,15]. A previous study showed that the overall resistance rates of NTS to cefotaxime and ceftriaxone were 1.7% and 1.1%, respectively [27]. The resistance rate to cefotaxime increased from 0.2% between 1996 and 2006 to 2.7% between 2007 and 2016. Similarly, the resistance rate to ceftriaxone increased from 0.9% between 1996 and 2006 to 1.5% between 2007 and 2016 [26]. In this study, a total of 51 (63.8%) of the 80 admitted children were administered an intravenous third-generation cephalosporin as the empiric antibiotic regimen. All children recovered without any complications despite the varying durations of antibiotic therapy.

This study had some limitations. First, only emergency room patients from a single medical center in central Taiwan were enrolled over 1 year. Hence, the study’s findings may not fully represent the presentation of pediatric acute infectious diarrhea in the ED. Second, this study specifically focused on children clinically diagnosed with conditions with bacterial pathogens. However, it is important to note that not all diagnoses were confirmed through stool culture or multiplex polymerase chain reaction results. Third, challenges were encountered while obtaining blood samples, resulting in missing laboratory data points. Finally, the initial study design was conducted using the VSS score, and the modified VSS score was not used in this current study and thus, might yield different interpretations. This factor may have introduced bias in our study. Fourth, detailed information on the decision-making process for admission arrangements and antibiotic prescriptions was not adequately recorded. Such limitations may have caused bias in analyzing pediatric acute infectious diarrhea in the ED.

## 5. Conclusions

Notably, a significant proportion of patients clinically diagnosed with acute febrile bacterial colitis by ED physicians were <6 years of age, and most of them required admission for further treatment. The primary causative organism identified was NTS. Age and elevated serum CRP or PCT levels are important factors that ED physicians should consider when determining whether a patient should be admitted. Third-generation cephalosporins such as ceftriaxone or cefotaxime are commonly recommended as the initial choice for empirical treatment in managing hospitalized children with suspected serious bacterial colitis.

## Figures and Tables

**Table 1:** Demographics and clinical presentations of the patients with acute infectious diarrhea.

Variables				Age (Years)						
		Total (n = 105)		≤1 (n = 21)		2–6 (n = 68)		≥7 (n = 16)		*p*-Value
		N	%	N	%	N	%	N	%	
Sex	Male	70	66.7	12	57.1	47	69.1	11	68.8	0.585
	Female	35	33.3	9	42.9	21	30.9	5	31.3	
Chillness	Yes	37	35.2	3	14.3	25	36.8	9	56.3	0.027 *
Abdominal pain	Yes	44	41.9	1	4.8	31	45.6	12	75.0	<0.001 *
Headache	Yes	11	10.5	0	0.0	2	2.9	9	56.3	<0.001 *
Muscle soreness	Yes	8	7.6	0	0.0	3	4.4	5	31.3	0.004 *
Gross blood in stool	Yes	42	40.0	14	66.7	28	41.2	0	0.0	<0.001 *
Gross mucus in stool	Yes	60	57.1	15	71.4	43	63.2	2	12.5	<0.001 *
Vomiting	Yes	49	46.7	6	28.6	32	47.1	11	68.8	0.052
Underlying disease	Yes	2	1.9	1	4.8	1	1.5	0	0.0	0.583
Contact history	Yes	17	16.2	3	14.3	12	17.6	2	12.5	1.000
Admission unit	OU	25	23.8	4	19.0	12	17.6	9	56.3	0.004 *
	Ward	80	76.2	17	81.0	56	82.4	7	43.8	

* Statistically significant by the χ^2^ or Fisher’s exact tests when appropriate. OU: observation unit.

**Table 2:** Comparison of laboratory tests and management of pediatric acute infectious diarrhea between the age groups.

Variables	Age (Years)										
		≤1			2–6			≥7			
	N	Mean	SD	N	Mean	SD	N	Mean	SD	*p*-Value ^a^	*p*-Value ^b^
Fever until ER visit (day)	21	1.9	1.5	68	2.6	1.3	16	1.9	1.0	0.013 *	0.468
Maximum body temperature (°C)	21	39.1	0.8	68	39.4	0.7	16	39.5	0.6	0.134	0.077
Diarrhea until ER visit (day)	21	2.0	1.3	68	2.5	1.5	16	1.6	1.1	0.027 *	0.549
Diarrhea frequency (times/day)	21	6.1	2.7	68	7.3	3.5	16	6.3	2.7	0.444	0.691
WBC (×10^9^/L)	21	11.3	5.5	66	9.1	4.1	16	10.3	4.3	0.119	0.591
Neutrophils (%)	21	53.1	11.0	66	55.8	16.7	16	81.0	10.2	<0.001 *	<0.001 *
Lymphocyte (%)	21	33.2	9.3	66	30.3	14.3	16	10.5	6.4	<0.001 *	<0.001 *
Hemoglobin (mg/dL)	21	11.7	1.1	66	12.2	1.0	16	13.0	0.9	<0.001 *	<0.001 *
Platelet count (×10^9^/L)	21	321.4	110.3	66	277.3	76.5	16	231.3	40.3	0.006 *	0.001 *
CRP (mg/L)	21	33.0	27.0	68	64.2	59.3	16	62.0	5.4	0.093	0.072
PCT (ng/mL)	21	2.7	10.1	65	3.2	7.6	15	2.1	5.6	0.177	0.312
Sodium (mmol/L)	21	135.6	30.	68	135.5	2.6	16	136.3	2.6	0.681	0.799
Potassium (mmol/L)	21	4.4	0.5	67	4.2	0.5	16	3.7	0.4	<0.001 *	<0.001 *
Glucose (mg/dL)	21	98.5	10.7	66	96.7	16.9	16	116.7	22.0	0.004 *	0.024 *
Lactate (mmol/L)	14	14.8	5.9	39	13.8	7.6	11	10.9	2.8	0.200	0.076
Stool routine (WBC count/HPF)	21	11.7	29.6	62	7.3	19.6	9	36.9	48.0	0.112	0.391
Duration of antibiotics therapy	8	5.4	2.3	38	4.4	1.9	6	4.2	2.9	0.172	0.058
Fever after admission (day)	17	2.4	1.1	56	2.5	1.9	7	2.4	0.5	0.512	0.491
Observation stays (hour)	4	4.3	3.2	12	5.8	3.1	9	4.8	2.2	0.516	0.855
Hospital stays (day)	21	4.1	2.7	68	4.0	2.3	16	2.7	2.5	0.071	0.115

WBC: white blood count, CRP: C-reactive protein, PCT: procalcitonin, HPF: high power field. * Statistically significant *p*-value ^a^ by the Kruskal–Wallis test or *p*-value ^b^ by the Jonckheere-Terpstra test.

**Table 3:** Comparison of clinical presentations and laboratory tests between hospitalized and no-hospitalized patients.

Variables	Admission						
	No			Yes			*p*-Value
	N	Mean	SD	N	Mean	SD	
Age (years)	25	5.9	4.6	80	3.1	2.8	0.007 *
Fever until ER visit (day)	25	2.1	1.2	80	2.4	1.4	0.323
Maximum body temperature (°C)	25	39.4	0.7	80	39.4	0.7	0.903
Diarrhea until ER visit (day)	25	1.99	1.2	80	2.4	1.6	0.097
Diarrhea frequency (times/day)	25	6.7	3.2	80	7.0	3.3	0.770
WBC (×10^9^/L)	23	8.8	3.9	80	10.0	4.7	0.217
Segment (%)	23	66.6	17.1	80	57.1	17.1	0.037 *
Lymphocyte (%)	23	21.8	14.3	80	29.5	14.2	0.028 *
Hemoglobin (mg/dL)	23	12.5	1.1	80	12.1	1.0	0.134
Platelet count (×10^9^/L)	23	270.3	90.5	80	281.7	82.6	0.285
CRP (mg/L)	25	25.5	22.2	80	67.6	57.8	<0.001 *
PCT (ng/mL)	23	1.2	4.6	78	3.4	8.5	<0.001 *
Sodium (mmol/L)	25	136.6	1.9	80	135.4	2.8	0.067
Potassium (mmol/L)	24	4.0	0.4	80	4.2	0.5	0.023 *
Glucose (mg/dL)	24	100.7	13.3	79	100.0	19.4	0.737
Lactate (mmol/L)	21	11.4	3.2	43	14.5	7.7	0.116
Stool routine (WBC count/HPF)	16	15.2	33.7	76	10.3	25.5	0.747

WBC: white blood count, CRP: C-reactive protein, PCT: procalcitonin, HPF: high power field. * Statistically significant by the Mann–Whitney U test.

**Table 4:** Multivariate logistic regression analysis of predictive factors associated with need for admission.

		Total	Admission, n (%)	Odds Ratio	95% CI			*p*-Value
Age	Median (IQR)	2 (2–4)	2 (2–3)	0.635	0.492	-	0.818	<0.001 *
CRP	Median (IQR)	3.82 (1.84–7.64)	5.27 (2.5–10.14)	1.404	1.044	-	1.887	0.025 *
PCT	<0.5	38	32 (82.4)	1.000				
	≥0.5	63	46 (73)	13.455	1.086	-	166.621	0.043 *

CI: confidence interval, IQR: interquartile range, CRP: C-reactive protein, PCT: procalcitonin. * Statistically significant.

**Table 5:** Comparison of laboratory tests and hospital stays of admitted patients with antibiotics treatment or not.

Variables	Antibiotics Treatment						
	No			Yes			*p*-Value
	N	Mean	SD	N	Mean	SD	
Age (years)	29	2.7	2.6	51	3.23	3.0	0.124
Fever until ER visit (day)	29	2.5	1.5	51	2.4	1.4	0.698
Maximum body temperature (°C)	29	39.3	0.7	51	39.4	0.7	0.533
Diarrhea until ER visit (day)	29	2.6	1.8	51	2.3	1.4	0.836
Diarrhea frequency (times/day)	29	7.7	4.0	51	6.6	2.8	0.359
WBC (×10^9^/L)	29	9.7	3.6	51	10.2	5.2	0.693
Segment (%)	29	52.1	16.5	51	59.8	16.9	0.063
Lymphocyte (%)	29	35.5	14.4	51	26.1	13.0	0.006 *
Hemoglobin (mg/dL)	29	12.1	1.2	51	12.1	0.9	0.707
Platelet count (×10^9^/L)	29	312.4	91.9	51	264.2	72.1	0.010 *
CRP (mg/L)	29	35.7	25.2	51	85.8	63.	<0.001 *
PCT (ng/mL)	28	0.6	0.5	50	5.1	10.3	<0.001 *
Sodium (mmol/L)	29	135.9	2.0	51	135.1	3.2	0.355
Potassium (mmol/L)	29	4.3	0.5	51	4.2	0.5	0.380
Glucose (mg/dL)	29	96.7	19.3	50	101.9	19.4	0.154
Lactate (mmol/L)	18	15.8	10.3	25	13.6	5.2	0.922
Stool routine (WBC count/HPF)	27	6.0	19.8	49	12.7	28.1	0.071
Hospital stays (day)	29	3.7	1.2	51	5.3	2.2	<0.001 *

WBC: white blood count, CRP: C-reactive protein, PCT: procalcitonin, HPF: high power field. * Statistically significant by the Mann–Whitney U test.

**Table 6:** Comparison of characteristics and associations of admitted children based on culture results of *Salmonella enteritidis*.

Variables				Culture Results of *Salmonella enteritidis*				
		Total (n = 80)		Negative (n = 52)		Positive (n = 28)		*p*-Value
		N	%	N	%	N	%	
Age (years)	≤1	17	21.3	9	17.3	8	28.6	0.082
	2–6	56	70.0	36	69.2	20	71.4	
	≥7	7	8.8	7	13.5	0	0.0	
Sex	Male	53	66.3	32	61.5	21	75.0	0.225
	Female	27	33.8	20	38.5	7	25.0	
Chillness	Yes	29	36.3	21	40.4	8	28.6	0.294
Abdominal pain	Yes	30	37.5	20	38.5	10	35.7	0.809
Headache	Yes	6	7.5	5	9.6	1	3.6	0.659
Muscle soreness	Yes	4	5.0	2	3.8	2	7.1	0.609
Gross blood in stool	Yes	37	46.3	19	36.5	18	64.3	0.018 *
Gross mucus in stool	Yes	55	68.8	31	59.6	24	85.7	0.016 *
Vomiting	Yes	37	46.3	25	48.1	12	42.9	0.655
Underlying disease	Yes	1	1.3	1	1.9	0	0.0	1.000
Contact history	Yes	14	17.5	8	15.4	6	21.4	0.545
PCT	<0.5	32	41.0	21	41.2	11	40.7	0.970
	≥0.5	46	59.0	30	58.8	16	59.3	
Blood culture	Negative	76	95.0	50	96.2	26	92.9	0.609
	Positive	4	5.0	2	3.8	2	7.1	
Stool routine (WBC/HPF)	No	45	59.2	29	60.4	16	57.1	0.779
	Yes	31	40.8	19	39.6	12	42.9	
Stool routine (OB)	No	32	42.1	19	39.6	13	46.4	0.560
	Yes	44	57.9	29	60.4	15	53.6	
Stool routine (Pus)	No	72	94.7	46	95.8	26	92.9	0.623
	Yes	4	5.3	2	4.2	2	7.1	
Antibiotic therapy	Yes	51	63.8	36	69.2	15	53.6	0.165
Duration of antibiotic therapy (day)	None	29	36.3	16	30.8	13	46.4	0.375
	1–3 days	18	22.5	13	25.0	5	17.9	
	>3 days	33	41.3	23	44.2	10	35.7	
Hospital stays (day)	≤3	24	30.0	16	30.8	8	28.6	0.838
	>3	56	70.0	36	69.2	20	71.4	
Hospital stays (day)	<5	53	66.3	39	75.0	14	50.0	0.024 *
	≥5	27	33.8	13	25.0	14	50.0	

PCT: procalcitonin, WBC: white blood count, HPF: high power field, OB: occult blood. * Statistically significant by the Chi-Square, or Fisher’s exact tests when appropriate.

## Data Availability

The data presented in this study are available on request from the corresponding author. The data are not publicly available due to privacy or ethical restrictions.
